# Impact of age of first exposure to *Plasmodium falciparum* on antibody responses to malaria in children: a randomized, controlled trial in Mozambique

**DOI:** 10.1186/1475-2875-13-121

**Published:** 2014-03-27

**Authors:** Augusto J Nhabomba, Caterina Guinovart, Alfons Jiménez, Maria N Manaca, Llorenç Quintó, Pau Cisteró, Ruth Aguilar, Arnoldo Barbosa, Mauricio H Rodríguez, Quique Bassat, John J Aponte, Alfredo Mayor, Chetan E Chitnis, Pedro L Alonso, Carlota Dobaño

**Affiliations:** 1 Centro de Investigação em Saúde de Manhiça (CISM), Maputo, Mozambique; 2 Barcelona Centre for International Health Research (CRESIB, Hospital Clínic - Universitat de Barcelona), Barcelona, Catalonia, Spain; 3 CIBER Epidemiología y Salud Pública (CIBERESP), Madrid, Spain; 4 International Centre for Genetic Engineering and Biotechnology (ICGEB), New Delhi, India

**Keywords:** Malaria, Antibodies, Acquired immunity, Age, Exposure, *Plasmodium falciparum*

## Abstract

**Background:**

The impact of the age of first *Plasmodium falciparum* infection on the rate of acquisition of immunity to malaria and on the immune correlates of protection has proven difficult to elucidate. A randomized, double-blind, placebo-controlled trial using monthly chemoprophylaxis with sulphadoxine-pyrimethamine plus artesunate was conducted to modify the age of first *P. falciparum* erythrocytic exposure in infancy and assess antibodies and malaria risk over two years.

**Methods:**

Participants (n = 349) were enrolled at birth to one of three groups: late exposure, early exposure and control group, and were followed up for malaria morbidity and immunological analyses at birth, 2.5, 5.5, 10.5, 15 and 24 months of age. Total IgG, IgG subclasses and IgM responses to MSP-1_19_, AMA-1, and EBA-175 were measured by ELISA, and IgG against variant antigens on the surface of infected erythrocytes by flow cytometry. Factors affecting antibody responses in relation to chemoprophylaxis and malaria incidence were evaluated.

**Results:**

Generally, antibody responses did not vary significantly between exposure groups except for levels of IgM to EBA-175, and seropositivity of IgG1 and IgG3 to MSP-1_19_. Previous and current malaria infections were strongly associated with increased IgG against MSP-1_19_, EBA-175 and AMA-1 (p < 0.0001). After adjusting for exposure, only higher levels of anti-EBA-175 IgG were significantly associated with reduced clinical malaria incidence (IRR 0.67, p = 0.0178).

**Conclusions:**

Overall, the age of first *P. falciparum* infection did not influence the magnitude and breadth of IgG responses, but previous exposure was critical for antibody acquisition. IgG responses to EBA-175 were the strongest correlate of protection against clinical malaria.

**Trial registration:**

ClinicalTrials.gov: NCT00231452.

## Background

Malaria is among the leading causes of morbidity and death in children, despite being a preventable disease. The World Health Organization estimates that 219 million cases and 660,000 deaths occurred in 2010, with approximately 81 and 91%, respectively, being in sub-Saharan Africa [1]. Under similar levels of exposure to *Plasmodium falciparum* different individuals experience different outcomes; while some may die of severe malaria others may experience mild disease to asymptomatic infection [2,3]. However, immunity to malaria is poorly understood. The rate of its development is thought to be associated with transmission intensity; it is both species and stage-specific and is rarely sterile [4]. In endemic areas, the acquisition of partial immunity to severe forms of the disease is observed relatively early in life [2], whereas immunity to parasitaemia and mild clinical malaria appear to take longer to develop and may require repeated parasite exposure for maintenance [5,6]. Human host genetics, parasite genetic variability and parasite-induced immunosuppression also influence the acquisition of immunity [7,8].

In people living in endemic areas, malaria infection induces strong humoral immune responses, involving production of predominantly immunoglobulin (Ig) M and IgG [9,10]. Cytophilic IgG isotypes, IgG1 and IgG3 are known to cooperate with monocytes to inhibit parasite growth *in vitro* by promoting phagocytosis of *P. falciparum*-infected erythrocytes [3,9,11,12]. Passive transfer of IgG from immune donors to non-immune children demonstrated that antibodies can be protective by reducing parasitaemia and clinical disease [13]. Parasite growth inhibition by plasma has been demonstrated *in vitro* for some blood stage antigens that are candidates for malaria vaccines. First, the recombinant 62 kDa apical membrane antigen-1 (AMA-1), involved in the re-orientation of merozoites prior to invasion of the erythrocyte [14]; second, the erythrocyte binding antigen (EBA-175), a 175 kDa protein assumed to bind glycophorin A (GpA) during invasion [14,15]; and, third, the 195 kDa merozoite surface protein-1 (MSP-1_19_), the most abundant merozoite surface protein, thought to be involved in the initial attachment of the merozoite to the erythrocyte surface [14]. Sero-epidemiological studies in populations of endemic areas have shown some evidences of the role of antibodies to these antigens in protecting against malaria [16].

Variant surface antigens (VSA) expressed on the parasitized erythrocyte membrane are thought to induce protective responses to *P. falciparum* as well [17]. The predominant VSA are encoded by ~60 var genes per parasite genome [18]. The gene product, called *P. falciparum* erythrocyte membrane protein 1 (PfEMP-1), is highly variant and equipped with several binding sites mediating adhesion of infected erythrocytes to vascular endothelium of capillaries and post-capillary venules thus avoiding destruction in the spleen [18-21]. Following acute disease, children develop specific immune responses to the repertoire of VSAs of the parasites that cause the infection [5,6]. Anti-VSA antibodies carried by the host at the time of disease impose a selection pressure on the repertoire of VSAs expressed during an infection [22].

Longitudinal studies in Indonesia [23] and Tanzania [24,25] suggested that a more mature immune system should acquire immunity against malaria more efficiently than a developing immune system, and that the age of first exposure to the parasite might influence the development of immunity. However, the exact effect of age of first exposure on the development of immunity was not clear, and this is critical for preventing children from avoidable life-threatening infections. Following conclusions from these studies, it was hypothesized that exposure to *P. falciparum* during the first six months of life was not relevant for acquisition of immunity, whereas exposure in the second six months could be critical for acquisition of immunity, and conducted a clinical trial to test this. The age of first exposure to *P. falciparum* blood stage infection was controlled by chemoprophylaxis with sulphadoxine-pyrimethamine (SP) plus artesunate (AS) during different periods of the first year of life in Mozambican children who were then followed up to 24 months for clinical malaria [26]. The conclusion of this trial was that after significantly interfering with exposure during the first year of life, the age of first exposure to malaria did not seem to affect the incidence of clinical malaria in the second year. However it remains to be established whether the intervention affected the acquisition of antibody responses to antigens considered to be targets of blood stage immunity.

A detailed assessment of antibody responses to MSP-1_19_, AMA-1, EBA-175 and VSA antigens was conducted in the children participating in the clinical trial during their first two years of life. Risk factors affecting antibody levels were examined at five cross sectional visits and upon *P. falciparum* infection and convalescence, and the association between the magnitude and breadth of antibody response and subsequent incidence of malaria.

## Methods

### Study site and population

The study was conducted in the District of Manhiça (Maputo Province) in Southern Mozambique, in the neighbourhoods of Maragra and Maciana in the town of Manhiça. Characteristics of the area have been described elsewhere [27]. Briefly, transmission of *P. falciparum* malaria is perennial with two well-demarked seasons, a dry season (May-October) and a rainy season (November-April). The entomological inoculation rate estimated in 2002 was of 38 infective bites/person/year [28]. A demographic surveillance system (DSS) that has been in place since mid-1996 continuously monitored the residents. Inhabitants are mostly subsistence farmers living in compounds separated by garden plots and grazing land [26].

### Study design

The study was a double-blind, randomized, placebo-controlled trial, described in detail elsewhere [26]. Briefly, 391 HIV-negative pregnant women were invited to have their infants participate in the trial. Forty-five newborns were excluded from the study: ten withdrawals, eight stillbirths, three deaths before inclusion, one unknown and twenty three who did not meet inclusion criteria (birth weight <2 kg, same gender twins, congenital malformations or birth asphyxia). A total of 349 newborns and their mothers were enrolled in the study and 287 children completed the two-year follow up [26]. Eligible newborns were randomized to one of three chemoprophylactic groups. The late exposure group received monthly chemoprophylaxis with SP + AS from 2.5 until 4.5 months of age and placebo from 5.5 to 9.5 months of age. The early exposure group received placebo from 2.5 until 4.5 months of age and monthly chemoprophylaxis from 5.5 to 9.5 months of age. The control group received monthly placebo from 2.5 to 9.5 months of age. Given the relatively long half-life of SP, it was assumed that the chemoprophylactic effect lasted for at least one month after each administration. One-and-a-half mL of capillary blood was collected for serological and parasitological determinations at cross-sectional visits (2.5, 5.5, 10.5, 15 and 24 months). Capillary blood from the first acute malaria infection and at convalescence, one month after the first malaria episode, was also collected for serological and parasitological analyses.

### Ethical clearance

Approval for the study was obtained from the National Mozambican Ethics Review Committee (Ref 05/CNBS/05) and the Hospital Clínic of Barcelona Ethics Review Committee. Children were enrolled in the study after their mothers gave a written informed consent.

### Parasite isolates, *in vitro* culture and measurement of IgG against VSA

In previous work by the same group, a panel of parasite isolates (Moz2, R29, 3D7, ItG and EB8) was tested for immunogenicity. The laboratory line (R29) was found to be the most immunogenic in the study area [29,30]. Stocks of ring stage at 3% parasitaemia were prepared from R29 *P. falciparum* parasite *in vitro* cultures. Parasites were cryopreserved by adding 1.7 volumes of glycerolyte dropwise and at a continuous agitation, and then kept at -80°C. For recovery, parasite aliquots were thawed using two sodium chloride thawing solutions (0.2 V of 12% NaCl and 10 V of 1.6%; and washed with RPMI incomplete media) following the protocol described elsewhere [6]. Parasite culture to late trophozoite was done using standard protocols [31].

The protocol for quantifying IgG against *P. falciparum* VSA has been described elsewhere [6]. Briefly, 95 μL of trophozoite-infected erythrocytes (T-iE) at 1% parasitaemia were sequentially exposed to 5 μL of plasma, 0.5 μL of polyclonal rabbit anti-human IgG (Dako, Glostrup, Denmark) and 0.1 μL of a goat anti-rabbit IgG antibody (Invitrogen, Molecular Probes, Carlsbad, USA) together with 0.1 μL of ethidium bromide (Applichen UN2810 1%). Samples were washed three times with PBS-1% BSA after each step of incubation with antibodies. Four serial dilutions (1/20, 1/40, 1/80, 1/160) of a pool of positive control plasmas (from healthy adults with life-long continuous exposure to *P. falciparum*) and one dilution (1/20) of five negative control plasmas (from adults without a history of contact with malaria parasites) were included in each plate. Samples were then acquired in a four-colour FACSCalibur (Becton Dickinson, USA) using CellQuest 3.3 software. Up to 1,000 T-iE (or ethidium bromide positive events) were counted per sample, and the geometric means (GM) of the fluorescence intensities were calculated.

### Enzyme-linked immunosorbent assay (ELISA)

All plasma samples were assayed for total IgG, isotypes (IgG1, IgG2, IgG3, IgG4) and IgM against the recombinant proteins MSP-1_19_ (19-kDa C-terminal fragment, 3D7 strain), AMA-1 (ectodomain, 3D7 strain), and EBA-175 (region II, fragment I, CAMP strain) from the ICGEB (New Delhi, India) [32]. Ninety-six-well plates were coated with 200 ng per well of antigen diluted in 0.05 M carbonate-bicarbonate buffer and incubated overnight at 4°C. Next day, plates were blocked with 2% bovine serum albumin in PBS-Tween for eight hours at 4°C. Then, plasma samples (dilution 1/200) were added in duplicates along with a positive control (a pool of plasmas from eight adults with lifelong exposure to malaria in the dilutions: 1/400, 1/800, 1/1,600 and 1/3,200), nine negative controls (plasmas from nine non-exposed adults, diluted 1/400) and two myeloma controls (for conjugated antibodies) 1 μg/ml and 0.5 μg/ml. All incubations were followed by three washings with 0.05% Tween 20 + BSA 0.25% in PBS. After one-hour incubation with 100 μL of peroxidase-conjugated rabbit anti-human IgG (1/8,000), IgM (1/3,000) (DakoCytomation) and sheep anti-human IgG1 (1/6,000), IgG2 (1/1,500), IgG3 (1/1,500), IgG4 (1/3,000) isotypes (Binding site, UK) secondary antibodies diluted in wash buffer, respectively, 100 μL of citric acid 0.243 M + Na_2_HPO_4_ 0.512 M + 1 mg/ml of o-phenylenediamine chromogen (Sigma, St Louis, MO, USA) with 0.012% H_2_O_2_ substrate were added per well for 5 min, and the colorimetric reaction was stopped with 25 μL of 3 M H_2_SO_4_. The specific reactivities of plasma samples were obtained as optical density (OD) values (absorbance measured at 492 nm normalized against a positive control run in every experiment). In addition, antibody levels were categorized using a cut-off OD value (the arithmetic mean of negative controls plus three standard deviations) for statistical analyses.

### Parasitaemia

*Plasmodium falciparum* infections were diagnosed and quantified by microscopy at 2.5 and 24 months of age, and in non-programmed visits whenever a child attended the hospital with fever or history of fever in the preceding 24 hours. Thick and thin blood films were Giemsa-stained and read as previously described [26]. Cases with submicroscopic parasitaemia were assessed by quantitative real time PCR (qPCR) performed from filter paper blood spots at birth (from cord blood), 2.5 and 24 months of age. DNA for qPCR analysis was extracted using the QIAamp DNA mini kit (Qiagen). The method used for qPCR was a minor modification of a protocol described elsewhere [33]. Briefly, the qPCR amplified the multicopy 18S ribosomal RNA gene of *P. falciparum*. The probe used was labelled with 6-carboxy-flourescein. A standard curve was produced using known concentrations of 3D7 strain *P. falciparum* parasites in parasite culture. Importing this curve into subsequent qPCR runs achieved quantification of parasitaemia with an ABI Prism 7500 apparatus (Applied Biosystems).

### Definitions and statistical methods

Clinical malaria was defined as axillary temperature ≥37.5°C or reported fever in the preceding 24 hours, plus positive parasitaemia by microscopy. For anti-VSAs antibodies the sample fluorescence intensity was defined as the difference between the GM of the fluorescence emitted by the T-iE and the GM of the fluorescence emitted by the non-infected erythrocytes. Antibody values for ELISA (ELISA - normalized OD values for total IgG, IgG isotypes and IgM) were logarithmically transformed and averaged within the groups and presented as GM plus 95% confidence intervals (CI). Distribution of antibody responses to each antigen at each cross-sectional visit, acute malaria and convalescence, are presented as weighted scatter plots. Differences in antibody prevalence (percentage of positive responders) between treatment groups at different time points were estimated by Chi-square and Fisher’s exact test, as appropriate, and differences in continuous antibody values among treatment groups were evaluated by ANOVA, evaluated using a likelihood ratio test and a global p-value was used for significance. Breadth of antibodies at the cross-sectional visits was defined as the number of antigens with positive IgG or IgM antibodies responses as defined by the categories above. Mixed-effects linear regression models with a random-intercept at the subject level were used to analyse factors independently associated with antibody levels at the cross-sectional visits, and the variables used to estimate models were: intervention group, age, season (dry/rainy), neighbourhood (grouped in 10 geographical categories), gender, previous infection, current infection, low birth weight, weight for age score, maternal peripheral infection, placental infection and inflammation, parity, maternal age, maternal anaemia, congenital infection (parasites in cord blood by qPCR), use of insecticide-treated nets (ITN) and documented indoor residual spraying (IRS) in the household. Selection of the variables for the multivariable model was done through a forward-stepwise procedure, where the criterion for including a variable to the model was p-value ≤0.05 from the likelihood ratio test. In addition, mixed-effects linear regression models were used to analyse the effect on antibody responses of the following variables selected for the highest biological plausibility to influence Ig levels: intervention group, age, season, neighbourhood, previous infection, current infection, placental infection and inflammation, congenital infection and low birth weight. Antibody levels and breadth of response at the cross-sectional visits in relation to incidence of malaria were assessed by negative binomial regression models. First, the control group (no treatment) was evaluated in two risk intervals from 2.5 to 12 months and from 2.5 to 24 months. Second, all treatment groups were assessed in three risk intervals: from 5.5 to 24 months, from 10.5 to 24 months and from 15 to 24 months. The strength of the association between antibody levels or breadth and malaria risk was assessed first unadjusted and second after adjusting by treatment, age, season, neighbourhood, current infection, previous infection, maternal infection, congenital infection, placental inflammation, ITN use and IRS. Statistical significance was defined at P < 0.05. Crude p values reported in this exploratory study were not adjusted for multiple comparisons and were interpreted for internal coherence, consistency of results and biological plausibility. Data analysis was performed using STATA 11 (StataCorp. 2007. Stata Statistical Software: Release 10. College Station, TX: StataCorp LP).

## Results

### Comparison of antibody responses between chemoprophylaxis groups

**Figure 1:**
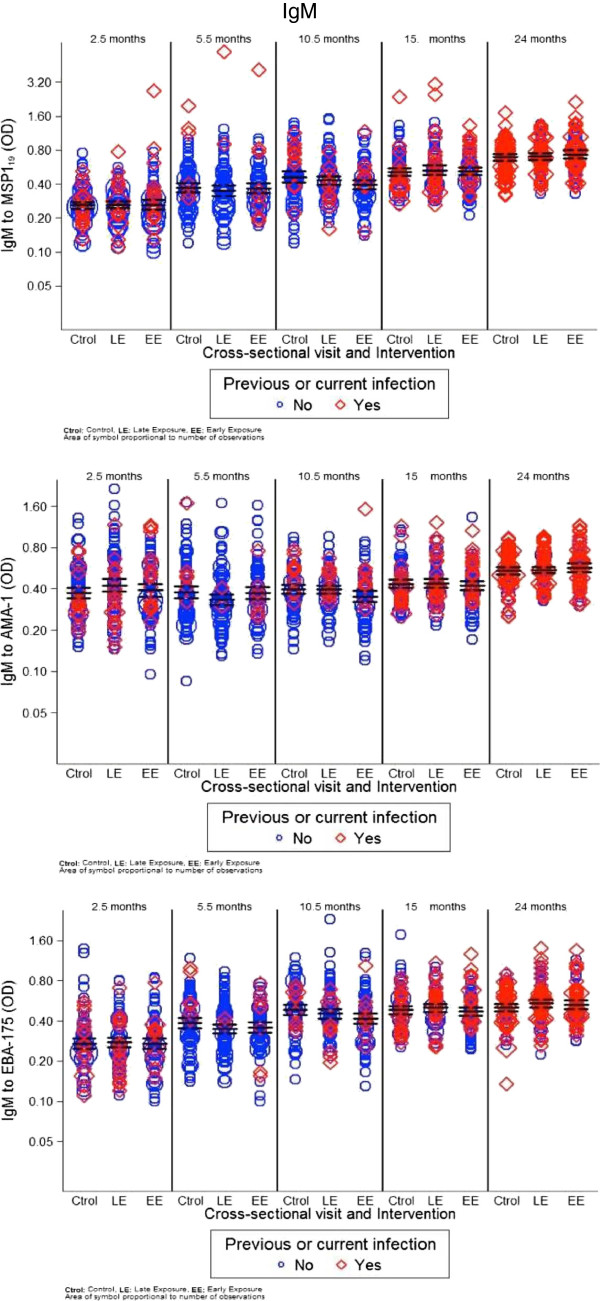
**Weighted scatter plots of IgM levels by cross-sectional visit and treatment group.** The area of the symbol is proportional to the number of observations. Red symbols correspond to IgM levels in those children with previous or current *Plasmodium falciparum* infection. Horizontal continuous and dashed lines indicate geometric means at 95% confidence interval.

**Figure 2:**
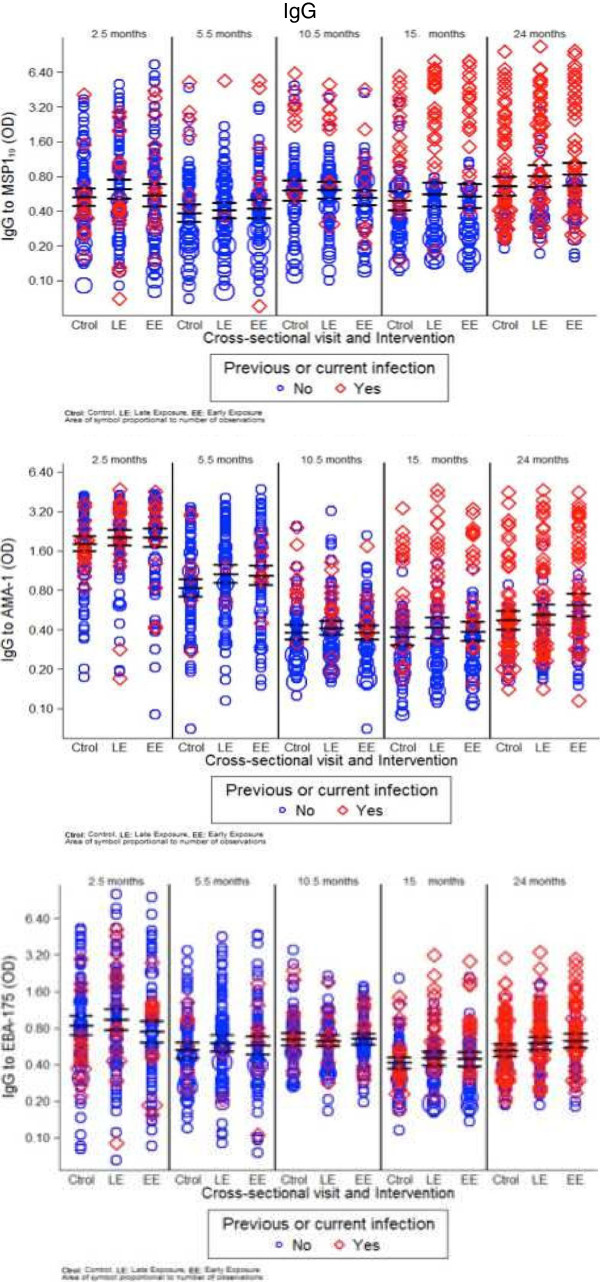
**Weighted scatter plots of IgG levels by cross-sectional visit and treatment group.** Red symbols correspond to IgG levels in those children with previous or current *Plasmodium falciparum* infection.

The magnitude of antibody responses was compared between study groups during the intervention period (first year) and after the intervention period (second year). Antibody responses to *P. falciparum* did not differ significantly between intervention arms at most study visits (cross-sectionals, first clinical acute episode or convalescence) for the majority of serological markers evaluated (Figures 1 and 2). Overall, results of linear regression analyses were similar after adjusting for previous episodes of clinical malaria.

In the first year of life, significant differences according to the intervention group were only observed in some antibody markers at month 10.5. Levels of IgM were higher in the late exposure and control groups, who were exposed to infection between months 5.5 and 10.5, compared to the early exposure group, who were under chemoprophylaxis during that period (GM, 95% CI; EBA-175: 0.45, 0.42-0.49 and 0.49, 0.45-0.53 *vs* 0.42, 0.38-0.46, respectively, p = 0.0469; AMA-1: 0.40, 0.37-0.43 and 0.40, 0.37-0.42 *vs* 0.35, 0.32-0.39, p = 0.0543; MSP-1_19_: 0.43, 0.39-0.47 and 0.46, 0.41-0.52 *vs* 0.40, 0.36-0.43, p = 0.0885) (Figure 1). Antibody levels did not differ between groups at month 5.5 following chemoprophylaxis in the late exposure group (administered at months 2.5 to 4.5). Geometric means and 95% CI for control, late exposure and early exposure groups were: MSP-1_19_: 0.37, 0.34-041; 0.35, 0.31-0.39 and 0.37, 0.33-0.41, p = 0.6894; AMA-1: 0.38, 0.34-0.42; 0.33, 0.31-0.37 and 0.37, 0.34-0.41, p = 0.1320; EBA-175: 0.39, 0.35-0.42; 0.35, 032–0.38 and 0.36, 0.33-0.39 p = 0.2106, respectively.

**Figure 3:**
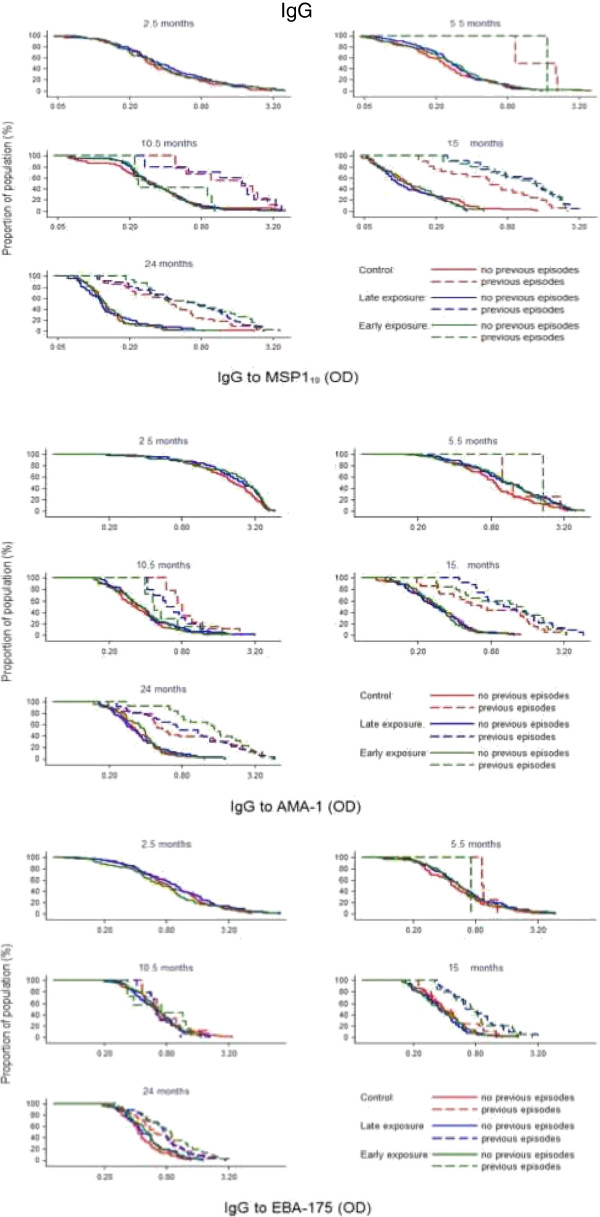
Reverse empirical distribution function of IgG levels by cross-sectional visit and treatment group in children stratified according to having (dashed line) or not (continuous line) previous episodes of clinical malaria.

In contrast, IgG levels did not significantly vary after chemoprophylaxis and thus there was no evidence for supporting differences in IgG levels between the three exposure groups at the cross-sectional visits (Figures 2 and 3). This was observed when comparing crude GM antibody levels, antibody prevalences, and also adjusted for previous clinical malaria episodes. The only instances where there were significant differences in IgG responses between intervention groups was also in the direction of lower IgG levels after drug administration, and this was manifested for cytophilic IgGs against MSP-1_19_ at month 10.5. Specifically, the prevalence of positive IgG1 responses was lower in the early exposure group compared to the late exposure and control groups (13 *vs* 28%, 28%, respectively; Chi^2^ p = 0.0248), and similarly for levels of IgG3 (linear regression model unadjusted p = 0.0284, adjusted for prior malaria p = 0.0343). Non-cytophilic antibodies (IgG2 and IgG4) yielded consistently low level responses and are not reported here.

**Figure 4:**
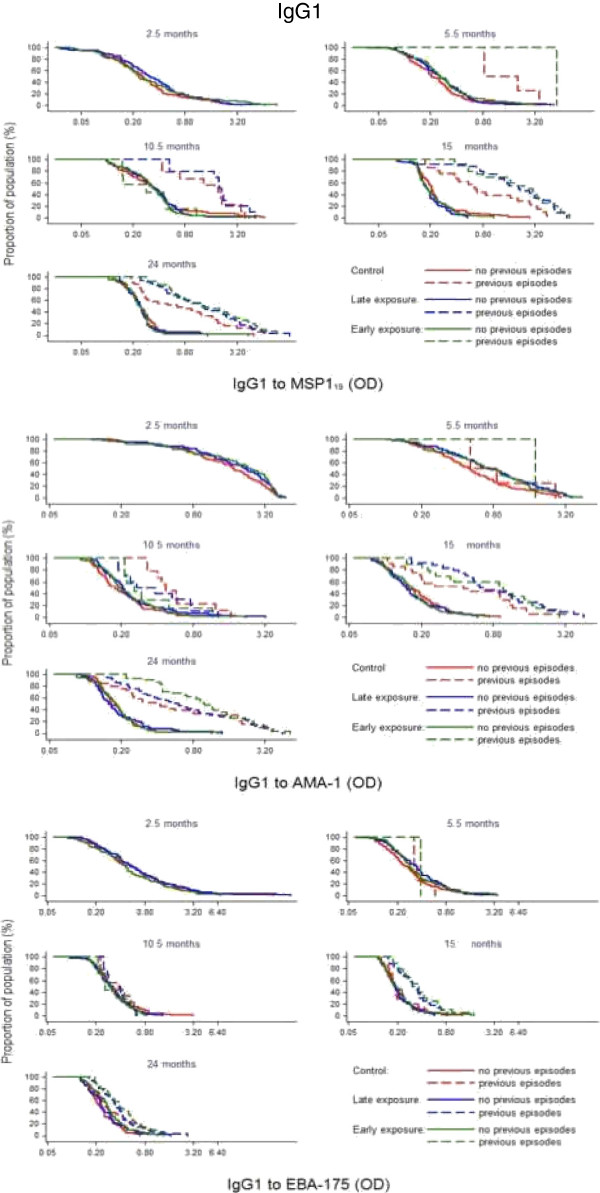
Reverse empirical distribution function of IgG1 levels by cross-sectional visit and treatment group in children stratified according to having (dashed line) or not (continuous line) previous episodes of clinical malaria.

**Figure 5:**
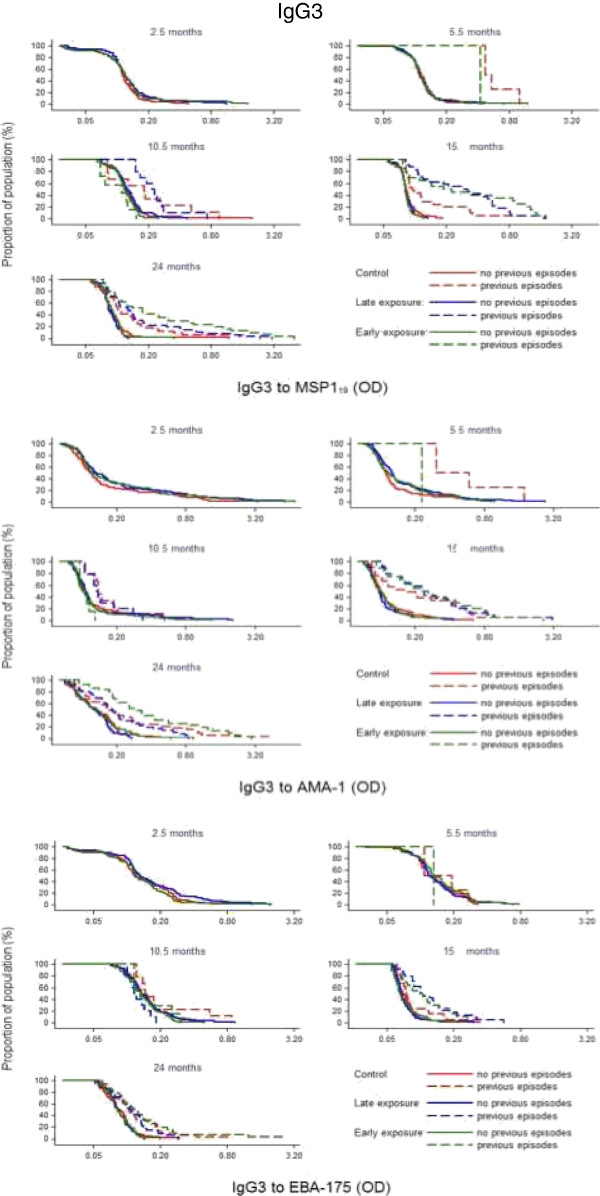
Reverse empirical distribution function of IgG3 levels by cross-sectional visit and treatment group in children stratified according to having (dashed line) or not (continuous line) previous episodes of clinical malaria.

In the second year, no significant differences in antibody levels were detected overall according to intervention group. However, children with prior malaria exposure (documented previous episodes of parasitologically confirmed clinical malaria) who had received SP + AS chemoprophylaxis any time during year 1 (early exposure and late exposure groups) had higher IgG to MSP-1_19_, EBA-175 and AMA-1 at 15 and 24 months of age than Control children who had been under continuous exposure to *P. falciparum* (Figures 3, 4 and 5). Regarding IgM responses, a higher seroprevalence for the early exposure group compared to the late exposure and control groups was also observed at age 24 months (MSP-1_19_: 13 *vs* 7% and 3%, respectively, p = 0.038; EBA-175: 6 *vs* 4% and 0%, p = 0.032; AMA-1: 26 *vs* 18% and 15%, p = 0.195; Chi^2^ test).

There were no statistically significant differences in IgG responses to VSA among the three groups at any of the sampling visits (Additional file 1).

### Analysis of factors affecting the magnitude of antibody responses

Age was the only factor significantly affecting the level of all antibodies against all antigens studied, even after adjusting for all the covariates (Table 1, p < 0.0001 and Additional file 2). IgM levels significantly increased with age (Figure 1). Overall, IgG levels were higher at month 2.5 presumably because of the antibodies acquired passively from the mother, then significantly decayed from 5.5 to 10.5 months, before a gradual increase onwards, depending on the antigen (Figure 2). Previous and current infections significantly increased IgG antibodies and isotypes against the majority of the studied antigens (p values between <0.0389 and 0.0001). Dry season was significantly associated with reduced levels of IgG to MSP-1_19_ and IgG1 to AMA-1 and EBA-175, and with higher levels of IgG3 to EBA-175 (Table 1, Additional file 2). Neighbourhood, reflecting heterogeneity in exposure to malaria infection, influenced levels of IgG, IgG1 and IgG3 mainly against MSP-1_19_ and AMA-1 (Table 1, Additional file 2). Particular neighbourhoods were consistently associated with higher antibody responses while other were neutral (Additional files 3 and 4). Other factors examined related to in utero parasite exposure such as placental infection or inflammation, congenital infection or low birth weight did not always affect all antibody levels significantly (Table 1, Additional file 2), but overall, parasite exposure variables that affected the magnitude of antibodies were similar between the stepwise and multilevel analyses. Gender and parity did not have an effect on any of the antibodies studied.

**Table 1:** Factors independently associated with the magnitude of antibody response by linear regression stepwise analysis

**Antibody**	**Antigen**	**Variable**	**Prop. diff**^**1**^	**95% CI**	**P value**^**2**^
IgG	MSP-1_19_	Previous infections	1.61	1.41; 1.84	< 0.0001
		Current infections	1.39	1.74; 1.64	0.0001
		Dry season	0.88	0.80; 0.97	0.0084
	AMA-1	Previous infections	1.25	1.12; 1.40	0.0001
		Current infections	1.23	1.08; 1.39	0.0019
		Dry season	0.90	0.84; 0.97	0.005
	EBA-175	Current infections	1.13	1.01; 1.27	0.0389
		Congenital infections	1.29	1.10; 1.52	0.0017
IgG1	MSP-1_19_	Previous infections	1.69	1.47; 1.95	0.0001
		Current infections	1.31	1.18; 1.68	0.0002
		Low birth weight	1.31	1.06; 1.63	0.0143
	AMA-1	Previous infections	1.26	1.11; 1.43	0.0003
		Current infections	1.29	1.11; 1.49	0.0007
		Dry season	0.86	0.79; 0.93	0.0003
		Placental infection	1.22	1.04; 1.43	0.0144
		Congenital infections	1.31	1.06; 1.62	0.0128
	EBA-175	Previous infections	1.13	1.03; 1.59	0.0105
		Congenital infections	1.34	1.13; 1.24	0.0008
		Dry season	0.88	0.82; 0.95	0.0008
IgG3	MSP-1_19_	Previous infections	1.14	1.06; 1.24	0.0009
		Current infections	1.54	1.38; 1.71	<0.0001
		Intervention	1.11	1.03; 1.20	0.0068
		Congenital infections	1.16	1.03; 1.30	0.0146
	AMA-1	Previous infections	1.16	1.04; 1.29	0.0066
		Current infections	1.34	1.17; 1.52	<0.0001
		Placental inflammation	1.27	1.06; 1.53	0.0099
	EBA-175	Current infections	1.14	1.04; 1.24	0.0008
		Dry season	1.10	1.04; 1.16	0.0008
IgM	MSP-1_19_	Current infections	1.16	1.09; 1.25	<0.0001
		Dry season	1.1	1.05; 1.15	<0.0001
	AMA-1	Dry season	0.90	0.86; 0.94	<0.0001
	EBA-175	Dry season	1.07	1.03; 1.12	0.0009

Models were adjusted for age and neighbourhood, which were also significantly associated with antibody levels (p < 0.0006).

^1^The proportional difference for a particular factor is the ratio between the average levels of antibodies in children with the factor *vs* children without the factor, adjusted for other variables in the model.

^2^P value using likelihood ratio test.

### Antibody responses during a first acute clinical malaria episode and at convalescence

**Figure 6:**
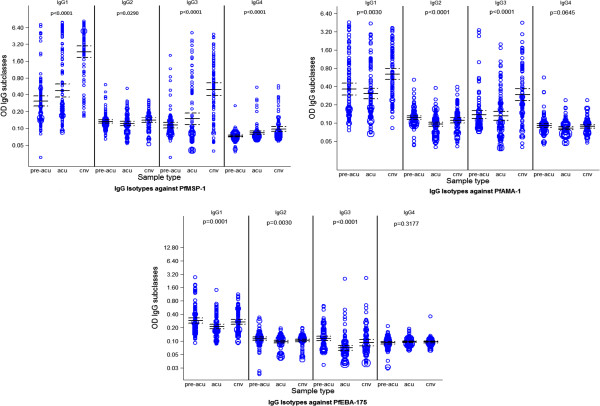
**Weighted scatter plot of antibody levels by type of sample before, during and after a first malaria clinical episode (pre-acu: pre-acute sample, acu: acute sample, cnv: convalescent sample).** The area of the symbol is proportional to the number of observations. Horizontal continuous and dashed lines indicate geometric means at 95% confidence intervals.

Antibody levels significantly varied following a first episode of clinical malaria compared to the levels measured at the preceding cross-sectional visits (Table 2). The most marked increases were observed from acute disease to convalescence for AMA-1 and MSP-1_19_ responses, and to a lesser extent for EBA-175 responses. The pattern of cytophilic IgG1 and IgG3 responses before, during and after a clinical malaria episode is shown in Figure 6.

**Table 2:** Levels of antibodies at the first episode of clinical malaria (acute) and one month later (convalescence) in relation to the levels of antibodies at the immediately preceding cross-sectional visit (pre-acute) in the three study groups

**Antibody**	**Antigen**	**Pre-acute**	**Acute**			**Convalescent**			
		**GM**^**1**^	**GM**	**Prop. diff.**^**2**^	**95% CI**	**GM**	**Prop. diff.**	**95% CI**	**P value**^**3**^
IgG	MSP-1_19_	0.47	0.73	1.56	1.20; 2.02	3.02	6.39	4.86; 8.40	< 0.0001
	AMA-1	0.56	0.50	0.88	0.71; 1.09	1.01	1.80	1.44; 2.25	< 0.0001
	EBA-175	0.53	0.41	0.77	0.65; 0.91	0.56	1.04	0.87; 1.24	< 0.0013
	VSA	17.46	8.60	0.49	0.27; 0.91	11.58	0.64	0.39; 1.06	< 0.0398
IgM	MSP-1_19_	0.45	0.68	1.50	1.29; 1.73	0.67	1.47	1.26; 1.72	< 0.0001
	AMA-1	0.39	0.44	1.11	0.99; 1.26	0.48	1.23	1.08; 1.39	< 0.0083
	EBA-175	0.42	0.49	1.14	1.03; 1.26	0.51	1.18	1.06; 1.31	< 0.0046
IgG1	MSP-1_19_	0.30	0.47	1.54	1.16; 2.04	2.32	7.48	5.54; 10.1	< 0.0001
	AMA-1	0.37	0.31	0.82	0.65; 1.04	0.64	1.73	1.35; 2.22	< 0.0001
	EBA-175	0.31	0.21	0.69	0.59; 0.81	0.27	0.87	0.74; 1.03	< 0.0001
IgG3	MSP-1_19_	0.11	0.15	1.30	0.99; 1.70	0.49	4.36	3.28; 5.78	< 0.0001
	AMA-1	0.14	0.13	0.93	0.77; 1.12	0.29	2.04	1.66; 2.50	< 0.0001
	EBA-175	0.11	0.07	0.61	0.52; 0.72	0.09	0.80	0.67; 0.95	< 0.0001

^1^Geometric means.

^2^The proportional difference is the ratio between the average levels of antibodies from acute (or convalescent) samples *vs* the levels from pre-acute samples, taking into account repeated measures for children.

^3^P value from Mixed-effects linear regression models using Likelihood Ratio Test.

### Antibody levels in relation to the incidence of clinical malaria

Two different sets of analyses were conducted at two different time risk intervals: from 2.5 months up to 12 months for the control group only, and from 5.5 months up to 24 months for all groups in the three time periods indicated in Tables 3, 4 and 5 (all children) and Additional file 5 (only control group). In general, no significant associations were found between the antibody levels and subsequent incidence of malaria for both unadjusted and adjusted analysis for the majority of antigens. In some cases, there was an association between higher IgM, IgG and/or IgG1 antibodies to AMA-1 and MSP-1_19_ and increased incidence of malaria (Tables 3, 4 and 5). The only response significantly associated with protection was IgG to EBA-175 whereby double amount of antibodies at 10.5 months correlated with reduced risk of clinical malaria up to 24 months of age follow up (IRR, 95% CI: 0.67, 0.47-0.94, p = 0.0178, adjusted) (Table 4).

**Table 3:** Association between levels of antibodies (two-fold increment) at 5.5 months of age and the incidence of malaria up to 24 months of age

		**5.5-24 months**					
**Antigens**	**Antibodies**	**Crude**			**Adjusted**		
		**IRR**^**1**^	**95% CI**	**P value**^**2**^	**IRR**	**95% CI**	**P value**
MSP-1_19_	IgG	1.09	0.90; 1.33	0.3783	0.96	0.78; 1.17	0.6853
	IgG1	1.10	0.90; 1.33	0.3525	0.96	0.79; 1.17	0.6872
	IgG2	1.33	0.72; 2.45	0.3594	1.05	0.61; 1.83	0.8519
	IgG3	1.20	0.87; 1.65	0.2508	0.93	0.67; 1.29	0.6599
	IgG4	0.66	0.27; 1.60	0.3526	0.48	0.21; 1.14	0.0970
	IgM	1.35	0.96; 1.89	0.0768	1.27	0.88; 1.85	0.2013
AMA-1	IgG	2.01	1.58; 2.56	< 0.0001	1.64	1.29; 2.09	< 0.0001
	IgG1	1.81	1.48; 2.22	< 0.0001	1.54	1.25; 1.89	< 0.0001
	IgG2	1.12	0.75; 1.66	0.5840	0.91	0.61; 1.36	0.6415
	IgG3	1.38	1.05; 1.82	0.0169	1.15	0.89; 1.47	0.2803
	IgG4	1.27	0.69; 2.33	0.4396	0.94	0.50; 1.78	0.8496
	IgM	1.33	0.89; 2.00	0.1590	1.16	0.79; 1.70	0.4468
EBA-175	IgG	1.29	1.04; 1.60	0.0177	1.11	0.90; 1.37	0.3323
	IgG1	1.36	1.08; 1.72	0.0077	1.21	0.97; 1.50	0.0916
	IgG2	1.21	0.69; 2.12	0.4981	1.21	0.72; 2.04	0.4614
	IgG3	1.34	0.97; 1.83	0.0701	1.20	0.89; 1.63	0.2324
	IgG4	0.91	0.45; 1.82	0.7837	1.09	0.57; 2.07	0.7936
	IgM	1.57	1.04; 2.38	0.0338	1.41	0.94; 2.12	0.0996
VSA	IgG	1.42	1.10; 1.84	0.0069	1.55	1.19; 2.01	0.0008

Analysis done by negative binomial regression models adjusted by treatment, season, neighbourhood, current infection, previous infection, maternal infection, congenital infection, placental inflammation, insecticide-treated bed net use, and indoor residual spraying.

^1^Incidence rate ratio.

^2^Negative binomial regression model using likelihood ratio test.

**Table 4:** Association between levels of antibodies (two-fold increment) at 10.5 months of age and the incidence of malaria up to 24 months of age

		**10.5-24 months**					
**Antigens**	**Antibodies**	**Crude**			**Adjusted**		
		**IRR**^**1**^	**95% CI**	**P value**^**2**^	**IRR**	**95% CI**	**P value**
MSP-1_19_	IgG	1.26	1.03; 1.55	0.0269	1.15	0.94; 1.40	0.1710
	IgG1	1.23	1.01; 1.49	0.0385	1.13	0.94; 1.36	0.2081
	IgG2	0.82	0.45; 1.49	0.5226	0.76	0.45; 1.27	0.2968
	IgG3	1.19	0.77; 1.82	0.4235	0.94	0.63; 1.39	0.7423
	IgG4	1.35	0.75; 2.42	0.3096	1.27	0.69; 2.33	0.4361
	IgM	0.98	0.65; 1.49	0.9419	1.01	0.70; 1.45	0.9654
AMA-1	IgG	1.86	1.37; 2.53	< 0.0001	1.31	1.00; 1.72	0.0533
	IgG1	1.68	1.26; 2.25	0.0002	1.21	0.94; 1.57	0.1366
	IgG2	1.12	0.57; 2.23	0.7446	0.98	0.57; 1.67	0.9354
	IgG3	1.06	0.71; 1.59	0.7446	0.98	0.71; 1.33	0.8743
	IgG4	2.78	1.13; 6.81	0.0261	1.85	0.83; 4.14	0.1360
	IgM	1.65	0.94; 2.87	0.0811	2.08	1.26; 3.45	0.0037
EBA-175	IgG	0.73	0.50; 1.09	0.1235	0.67	0.47; 0.94	0.0178
	IgG1	0.75	0.50; 1.13	0.1735	0.75	0.54; 1.04	0.0874
	IgG2	0.74	0.50; 1.09	0.1124	0.69	0.50; 0.90	0.0239
	IgG3	0.90	0.58; 1.40	0.6580	0.80	0.55; 1.17	0.2469
	IgG4	0.89	0.56; 1.41	0.6152	0.75	0.49; 1.14	0.1741
	IgM	1.12	0.72; 1.74	0.6113	1.48	1.02; 2.14	0.0358
VSA	IgG	1.02	0.76; 1.36	0.8827	0.92	0.68; 1.24	0.5864

Analysis done by negative binomial regression models adjusted by treatment, season, neighbourhood, current infection, previous infection, maternal infection, congenital infection, placental inflammation, insecticide-treated bed net use, and indoor residual spraying.

^1^Incidence rate ratio.

^2^Negative binomial regression model using likelihood ratio test.

**Table 5:** Association between levels of antibodies (two-fold increment) at 15 months of age and the incidence of malaria up to 24 months of age

		**15-24 months**					
**Antigens**	**Antibodies**	**Crude**			**Adjusted**		
		**IRR**^**1**^	**95% CI**	**P value**^**2**^	**IRR**	**95% CI**	**P value**
MSP-1_19_	IgG	1.56	1.33; 1.82	< 0.0001	1.29	1.06; 1.57	0.0121
	IgG1	1.62	1.39; 1.90	< 0.0001	1.37	1.13; 1.66	0.0011
	IgG2	2.14	1.07; 4.27	0.0260	1.37	0.71; 2.28	0.4141
	IgG3	1.84	1.36; 2.48	< 0.0001	1.32	0.98; 1.77	0.0591
	IgG4	2.22	0.79; 6.21	0.1033	1.35	0.60; 3.05	0.4694
	IgM	0.83	0.53; 1.31	0.4290	0.84	0.55; 1.27	0.4027
AMA-1	IgG	1.87	1.52; 2.30	< 0.0001	1.43	1.14; 1.79	0.0023
	IgG1	1.96	1.61; 2.38	< 0.0001	1.55	1.27; 1.90	< 0.0001
	IgG2	2.32	1.21; 4.44	0.0086	1.89	1.12; 3.20	0.0161
	IgG3	1.99	1.48,2.67	< 0.0001	1.41	1.08; 1.83	0.0113
	IgG4	2.55	1.24; 5.23	0.0088	1.71	0.95; 3.05	0.0723
	IgM	0.78	0.45; 1.37	0.3973	0.64	0.39; 1.04	0.0715
EBA-175	IgG	1.48	1.08; 2.04	0.0141	1.14	0.83; 1.57	0.4052
	IgG1	1.78	1.19; 2.68	0.0040	1.31	0.91; 1.89	0.1442
	IgG2	0.88	0.42; 1.82	0.7257	0.55	0.27; 1.10	0.0898
	IgG3	1.80	1.03; 3.15	0.0283	0.96	0.63; 1.48	0.8697
	IgG4	1.40	0.62; 3.17	0.4025	0.98	0.51; 1.89	0.9600
	IgM	0.65	0.36; 1.17	0.1510	0.72	0.42; 1.20	0.2133
VSA	IgG	1.68	1.18; 2.38	0.0034	1.29	0.94; 1.76	0.1105

Analysis done by negative binomial regression models adjusted by treatment, season, neighbourhood, current infection, previous infection, maternal infection, congenital infection, placental inflammation, insecticide-treated bed net use, and indoor residual spraying.

^1^Incidence rate ratio.

^2^Negative binomial regression model using likelihood ratio test.

### Antibody breadth of response in relation to the incidence of clinical malaria

Breadth of antibody responses in relation to malaria risk was determined only for total IgG and IgM against MSP-1_19_, AMA-1, EBA-175 and VSA in all groups (Table 6) and just control group (Additional file 6). Overall, there was an association between higher breadth of response and higher incidence of clinical malaria (IRR > 1 using 0–1 as reference group), which reached statistical significance for breadth of IgG responses at 15 months in relation to risk of malaria up to 24 months of age, in both crude and adjusted analyses for either all groups or the control group. The association between higher breadth of response and higher incidence of clinical malaria was also statistically significant at the risk interval between 5.5 months up to 24 months of age when including all children. The IRR values were higher and the p values more significant in crude than in the analyses adjusting for known past/current parasite exposure confounders. Regarding breadth of IgM antibody responses there was no statistically significant association in any risk intervals and group (Table 6, Additional file 6).

**Table 6:** Association between breadth of antibody response and the incidence of malaria in all chemoprophylactic groups up to 24 months of age

		**All groups**									
**Abs**^**1**^	**Time**	**Crude**					**Adjusted**				
		**Bth**^**2**^	**N**^**3**^	**IRR**^**4**^	**95% CI**	**P value**^**5**^	**Bth**	**N**	**IRR**	**95% CI**	**P value**
IgG	5.5-24 months	0-1	152	1	-	0.0005	0-1	152	1	-	0.0143
		2	47	3.50	1.79; 6.84		2	47	2.64	1.38; 5.06	
		3	9	3.17	0.82; 12.28		3	9	1.86	0.48; 7.24	
	10.5-24 months	0-1	189	1	-	0.3412	0-1	189	1	-	0.4438
		2	3	4.42	0.39; 49.95		2	3	0.89	0.09; 8.41	
		3	1	0.85	0.01; 75.19		3	1	11.72	0.29; 504.65	
	15-24 months	0-1	185	1	-	< 0.0001	0-1	185	1	-	0.0297
		2	19	8.56	3.68; 19.94		2	19	3.29	1.49; 7.29	
		3	5	5.42	1.11; 26.52		3	5	2.30	0.55; 9.54	
		4	1	13.68	0.48; 388.95		4	1	6.14	0.52; 71.92	
IgM	5.5-24 months	0	265	1	-	0.9316	0	265	1	-	0.1472
		1	23	1.04	1.04; 2.63		1	23	0.50	0.20; 1.26	
	10.5-24 months	0	246	1	-	0.2402	0	246	1	-	0.6655
		1	16	0.40	0.12; 1.35		1	16	1.22	0.41; 3.62	
		2	4	2.36	0.30; 18.24		2	4	1.91	0.40; 9.11	
	15-24 months	0	246	1	-	0.8912	0	246	1	-	0.7638
		1	14	1.00	0.28; 3.58		1	14	0.58	0.16; 2.09	
		2	5	2.08	0.29; 15.04		2	5	1.56	0.32; 7.57	
		3	1	0.97	0.01; 91.36		3	1	0.79	0.03; 19.06	

Analysis done by negative binomial regression. All chemoprophylactic groups were assessed in three risk intervals: from 5.5 months to 24 months, from 10.5 to 24 months and from 15.5 to 24 months. The strength of the association between breadth of response and malaria risk was assessed first unadjusted and after adjusting by treatment, age, season, neighbourhood, current infection, previous infection, maternal infection, congenital infection, placental inflammation, insecticide-treated bed net use, and indoor residual spraying.

^1^Antibody types.

^2^Breadth of antibodies.

^3^Number of children.

^4^Incidence rate ratio.

^5^Negative binomial regression model using likelihood ratio test.

## Discussion

This study found no evidence to support that the time of first exposure to blood stage *P. falciparum* infection, as controlled by SP + AS anti-malarial chemoprophylaxis, has a major influence in the acquisition of antibody responses to the MSP-1_19_, AMA-1, EBA-175 and VSA antigens examined. Consistent with the results of the clinical trial [26], the development of naturally acquired immunity within the parameters analysed (total IgM, IgG and subclasses) was not heavily impacted by a substantial disruption of parasite exposure. Significant decreases were only observed in IgM and IgG responses to some antigens in the early exposure group at month 10.5 following chemoprophylaxis, i.e., lower levels of IgM to EBA-175 and lower levels of positive IgG1 and IgG3 responses to MSP-1_19_. Thus, IgM to blood stage antigens at age 10.5 months appeared to be a surrogate of recent *P. falciparum* exposure. Maternal IgG levels were high at 2.5 months in all groups before waning over the first year. The conclusion of this first analysis was that, overall, a 3–5 month-long drug intervention had no major impact on *P. falciparum*-specific IgG levels in these young infants in face of high levels of maternal IgG antibodies still circulating in their plasma. Because of the confounding effect of these passively transferred antibodies, the impact of the study interventions on IgG levels during the first year of life and the impact of IgG responses during the first year of life on subsequent risk of malaria were difficult to ascertain.

Of note, children who received chemoprophylaxis during the first year of life and suffered previous malaria infections were characterized by an increase in antibody levels in the second year, as observed in the stratified cumulative distribution plots (Figures 3, 4 and 5). This pattern appeared to be parallel to the Kaplan Meier survival curves for cumulative proportion of children with at least one episode of malaria during the second year of follow-up, which showed a trend for a higher incidence of malaria in year 2 in the late and early exposure groups when compared to the control group [26]. Thus, these increased IgG antibody levels in children with documented parasite exposure reflected the higher incidence of malaria in the second year in those who received chemoprophylaxis in the first year. Therefore, antibodies to these blood stage antigens appeared to be mostly markers of exposure, and this is consistent with the strong association between previous or concurrent infection and IgG levels and breadth demonstrated here and in previous studies by our group [34,35]. Indeed, data show that high levels of antibody responses to antigens such as AMA-1 are commonly associated with greater future risk of malaria, presumably because children with increased antibodies levels or breadth are also those who have suffered more previous episodes of malaria and/or prenatal parasite exposure, a known and strong risk factor for subsequent malaria episodes [32,34,36]. This is in contrast to other studies showing an association between AMA-1 [3,16,37,38] or MSP-1_19_[4,16,39] responses and reduced risk of malaria. Even though, for example, anti-AMA-1 IgG antibodies are described as some times protective [3] while other non-protective [40], many studies fail to establish any significant association [41]. Dissimilarities between reports may be partially related to differences in age between participants, under two years old in this study *vs* older children or adults in other studies. However, a prominent factor that influence immune correlates analysis is heterogeneity in parasite exposure at the individual level. The challenges of establishing protective associations between antibody responses and malaria susceptibility if prior and present infections are not registered and taken into account in the analysis are increasingly being recognized. The only instance in which there was a protective association between higher levels of antibodies and reduced incidence of malaria was for the EBA-175 antigen, and this was mostly shown after analyses were adjusted for previous malaria episodes. Since this result has consistently been reproduced in two other studies in the same area with two different groups of children [35,42], it is concluded that among the antigen targets evaluated, EBA-175 is the most promising blood stage candidate for vaccine development.

Factors affecting the magnitude of anti-malarial antibodies were analysed in detail. Previous studies report that increasing age (particularly for AMA-1), heterogeneity of exposure, proximity to swamps, and higher previous exposure are significant predictors of higher antibody responses [4,32,35]. In this study, age was also the most prominent factor significantly influencing the levels of all antibodies. In addition, factors associated to concurrent or past parasite exposure, including during gestation and birth, generally affected antibody levels in these infants. Season and particular neighbourhoods had increased antibody prevalences, however it was not possible to define the geographic characteristics (e. g., proximity to swamps) explaining the differences. This study was limited in that it could not fully evaluate individual heterogeneity of exposure and differential antibody levels in relation to geographical parameters influencing malaria transmission intensity.

In these very young children, anti-VSA IgG responses were not prominent. Levels were very low and dependent on neighbourhood and age, with an overall pattern showing higher maternally derived IgG waning over time. This was in contrast to studies in a different endemic area of Tanzania where the level of antibodies increased with age from five to 24 months [22]. The results of this study do not support acquisition of VSA IgG by the age of two years in the Manhiça population at the time of the clinical trial. There was a lack of association between these antibodies and current malaria infection, in contrast to what was observed in another study conducted in the same area at a different time [29], and in Tanzania [22]. This suggests that in these children, anti-VSA IgG were not involved in early acquisition of immunity, presumably due to the high variability of PfEMP-1 antigens and the lower level of exposure during this limited time.

Breadth of antibody response has been suggested to be an important predictor of protection from clinical malaria in Kenyan children [9], with an inverse association between increasing breadth of IgG antibody specificities and risk of malaria, and none of the children who simultaneously had high antibody levels to five or more antigens experiencing a clinical episode. Studies assessing interaction between breadth and risk of malaria are limited. Here, using only four antigens, a direct association between breadth of IgG response and subsequent incidence of malaria was either not detected or observed at two different follow up periods (5.5-24 and 15–24 months of age), in contrast to the Kenyan study but in the same line as the relationship between magnitude of antibody responses and malaria risk. In Manhiça, the association between IgG antigenic breadth and incidence of malaria was stronger in the unadjusted analysis and weaker when controlling for variables related to parasite exposure, suggesting that it was highly affected by prior infection but that we not were able to remove all the confounders. Thus, in immune correlates analyses in mother-child cohorts it remains a challenge to account for absolute parasite exposures when using a primarily passive surveillance design, particularly to detect those infections that may be asymptomatic and not accounted for in the adjusted analyses.

## Conclusion

The age of first *P. falciparum* exposure seemed not to be critical for acquisition of protective immunity as evaluated by acquisition of antibody responses to blood stage antigens that are candidate targets for malaria vaccine development. Overall, the breadth and magnitude of antibody responses were strongly influenced by previous exposure and age. Only levels of IgG to EBA-175 were associated with reduced incidence of malaria in the second year of life. It remains to be assessed whether the intervention might have an impact on the quality of antibody responses such as affinity/avidity, fine specificity or functionality, and on cellular immune responses also thought to contribute to naturally acquired immunity.

## Competing interests

The authors declare that they have no competing interests.

## Authors’ contributions

PA, JJA and CD conceived and designed the field trial: CG, QB, AN, MNM, AB, and MHR conducted the field trial and follow-up of participants; CD and AM conceived and designed the experiments; CEC produced recombinant proteins; AN, AJ, MNM, RA, MHR, AB, and PC processed samples and performed laboratory analysis; LQ and JJA analysed the data; AN and CD wrote the paper. All authors contributed to the interpretation of the results and writing of the manuscript. All authors read and approved the final manuscript.

## Supplementary Material

Additional file 1 **Comparison of the anti-variant surface antigens (VSA) Immunoglobulin (Ig) G antibody responses.** Differences in anti-VSA IgG antibodies levels among chemoprophylaxis groups, at the five sampling study visits; assessed by ANOVA. Click here for file

Additional file 2 **Variables associated to antibody levels assessed by multilevel regression model.** Analyse of variables associated with antibody levels assessed by mixed-effects linear regression models including the following variables: intervention group, age, season, neighbourhood, previous infection, current infection, low birth weight, placental infection, placental inflammation and congenital infection. Click here for file

Additional file 3 **Association between different neighborhoods and antibody levels.** Proportional differences and P-values of the association between neighbourhood and the magnitude of antibody response by linear regression analysis. Click here for file

Additional file 4 **District map of the Manhiça study area.** Group of study regions and distribution of study participants per neighbourhood (bairros). Click here for file

Additional file 5 **Magnitude of antibody response related to incidence of clinical malaria only in control group.** Association between levels of antibodies (2-fold increment) at 2.5 months of age and the incidence of malaria up to 12 and 24 months of age, only for the placebo control group. Analysis done by negative binomial regression models adjusted by season, neighborhood, current infection, previous infection, maternal infection, congenital infection, placental inflammation, insecticide treated bednet use and indoor residual spraying. Click here for file

Additional file 6 **Breadth of antibody response related to incidence of clinical malaria only in control group.** Analysis done by negative binomial regression. Participants of control group were assessed in five risk intervals: from 2.5 months to 12 months, from 2.5 months to 24 months, from 5.5 months to 24 months, from 10.5 to 24 months and from 15.5 to 24 months. The strength of the association between breadth of response and malaria risk was assessed first unadjusted and after adjusting by treatment, age, season, neighbourhood, current infection, previous infection, maternal infection, congenital infection, placental inflammation, insecticide treated bednet use and indoor residual spraying. Click here for file
