# Supplementary material for: Impact of age of first exposure to Plasmodium falciparum on antibody responses to malaria in children: a randomized, controlled trial in Mozambique
Source: Malar J. 2014 Mar 27;13:121. doi: 10.1186/1475-2875-13-121 (PMC3986595; doi:10.1186/1475-2875-13-121)
Supplement: Additional file 1 — Comparison of the anti-variant surface antigens (VSA) Immunoglobulin (Ig) G antibody responses. Differences in anti-VSA IgG antibodies levels among chemoprophylaxis groups, at the five sampling study visits; assessed by ANOVA. [file 1475-2875-13-121-S1.doc]

Additional file 1

| Visits | Groups | IgG to VSA^2^ (OD^3^) | | P value^5^ |
| --- | --- | --- | --- | --- |
|  |  | GM^1^ | 95% CI^4^ |  |
| 2.5 months | Ctrl | 58.02 | 47.70; 70.57 | 0.4439 |
|  | LE | 69.03 | 54.54; 87.37 |  |
|  | EE | 69.52 | 53.73; 89.97 |  |
| 5.5 months | Ctrl | 19.13 | 16.10; 22.73 | 0.2197 |
|  | LE | 23.55 | 19.39; 28.60 |  |
|  | EE | 22.98 | 18.96; 27.86 |  |
| 10.5 months | Ctrl | 10.08 | 8.63; 11.76 | 0.5789 |
|  | LE | 11.20 | 9.76; 12.80 |  |
|  | EE | 10.58 | 9.21; 12.16 |  |
| 15 months | Ctrl | 11.03 | 9.61; 12.67 | 0.7586 |
|  | LE | 10.78 | 9.29; 12.51 |  |
|  | EE | 11.57 | 10.32; 12,98 |  |
| 24 months | Ctrl | 12.58 | 10.19; 15.54 | 0.7148 |
|  | LE | 12.68 | 10.97; 14.67 |  |
|  | EE | 13.87 | 11.52; 16.70 |  |

^1^ Geometric means

^2^ Variant surface antigens

^3^ Optical density

^4^ Confidence interval

^5^ P value using likelihood ratio test

Ctrl: control group; LE: late exposure groups; EE: early exposure group
