# Supplementary material for: Impact of age of first exposure to Plasmodium falciparum on antibody responses to malaria in children: a randomized, controlled trial in Mozambique
Source: Malar J. 2014 Mar 27;13:121. doi: 10.1186/1475-2875-13-121 (PMC3986595; doi:10.1186/1475-2875-13-121)
Supplement: Additional file 3 — Association between different neighborhoods and antibody levels. Proportional differences and P-values of the association between neighbourhood and the magnitude of antibody response by linear regression analysis. [file 1475-2875-13-121-S3.doc]

Additional file 3

| **Antibody** | **Antigen** | **Neighbourhood** | **P. diff^1^** | **95% CI^2^** | **P value^3^** | **Overall P*-*value** |
| --- | --- | --- | --- | --- | --- | --- |
| IgG | MSP-1_19_ | 2 | 0.92 | 0.70; 1.20 | 0.5262 | 0.0006 |
|  |  | 3 | 1.01 | 0.77; 1.32 | 0.9513 |  |
|  |  | 4 | 1.31 | 1.03; 1.66 | 0.0268 |  |
|  |  | 5 | 0.82 | 0.64; 1.04 | 0.1058 |  |
|  |  | 6 | 0.83 | 0.68; 1.01 | 0.0687 |  |
|  |  | 7,1 | 0.92 | 0.75; 1.13 | 0.4098 |  |
|  |  | 9 | 1.23 | 0.90; 1.68 | 0.2032 |  |
|  |  | Others | 0.81 | 0.49; 1.33 | 0.4039 |  |
|  | AMA-1 | 2 | 0.94 | 0.74; 1.19 | 0.5956 | <0.0001 |
|  |  | 3 | 0.76 | 0.60; 0.97 | 0.0276 |  |
|  |  | 4 | 1.21 | 0.98; 1.49 | 0.0791 |  |
|  |  | 5 | 0.71 | 0.57; 0.88 | 0.0021 |  |
|  |  | 6 | 0.66 | 0.56; 0.79 | < 0.0001 |  |
|  |  | 7,1 | 0.9 | 0.75; 1.08 | 0.2698 |  |
|  |  | 9 | 1.3 | 0.99; 1.72 | 0.0629 |  |
|  |  | Others | 0.82 | 0.55; 1.23 | 0.3331 |  |
|  | EBA-175 | 2 | 0.86 | 0.70; 1.07 | 0.1817 | 0.0006 |
|  |  | 3 | 0.95 | 0.76; 1.18 | 0.6424 |  |
|  |  | 4 | 1.17 | 0.97; 1.41 | 0.1032 |  |
|  |  | 5 | 0.85 | 0.70; 1.04 | 0.1136 |  |
|  |  | 6 | 0.8 | 0.68; 0.93 | 0.0051 |  |
|  |  | 7,1 | 0.9 | 0.76; 1.06 | 0.1982 |  |
|  |  | 9 | 0.92 | 0.71; 1.18 | 0.5151 |  |
| IgG1 | MSP-1_19_ | 2 | 0.9 | 0.68; 1.19 | 0.4623 | <0.0001 |
|  |  | 3 | 0.95 | 0.72; 1.26 | 0.7269 |  |
|  |  | 4 | 1.37 | 1.07; 1.76 | 0.0119 |  |
|  |  | 5 | 0.79 | 0.61; 1.02 | 0.0658 |  |
|  |  | 6 | 0.8 | 0.65; 0.99 | 0.0407 |  |
|  |  | 7,1 | 0.92 | 0.75; 1.14 | 0.4624 |  |
|  |  | 9 | 1.37 | 0.99; 1.91 | 0.0583 |  |
|  |  | Others | 0.92 | 0.55; 1.54 | 0.7473 |  |
|  | AMA-1 | 2 | 0.92 | 0.69; 1.22 | 0.5595 | <0.0001 |
|  |  | 3 | 0.78 | 0.59; 1.04 | 0.0875 |  |
|  |  | 4 | 1.18 | 0.92; 1.50 | 0.1883 |  |
|  |  | 5 | 0.65 | 0.50; 0.83 | 0.0008 |  |
|  |  | 6 | 0.62 | 0.50; 0.76 | < 0.0001 |  |
|  |  | 7,1 | 0.87 | 0.70; 1.07 | 0.1906 |  |
|  |  | 9 | 1.31 | 0.94; 1.81 | 0.1055 |  |
|  |  | Others | 0.83 | 0.52; 1.32 | 0.4315 |  |
| IgG3 | MSP-1_19_ | 2 | 1.06 | 0.90; 1.24 | 0.4825 | 0.0001 |
|  |  | 3 | 0.92 | 0.79; 1.08 | 0.3038 |  |
|  |  | 4 | 1.05 | 0.92; 1.21 | 0.469 |  |
|  |  | 5 | 0.81 | 0.70; 0.93 | 0.0035 |  |
|  |  | 6 | 0.86 | 0.76; 0.96 | 0.0087 |  |
|  |  | 7,1 | 0.87 | 0.77; 0.98 | 0.0201 |  |
|  |  | 9 | 1.03 | 0.86; 1.24 | 0.7525 |  |
|  |  | Others | 0.91 | 0.67; 1.23 | 0.5368 |  |
|  | AMA-1 | 2 | 1.13 | 0.91; 1.41 | 0.2793 | 0.0004 |
|  |  | 3 | 1.06 | 0.84; 1.32 | 0.6386 |  |
|  |  | 4 | 1.21 | 0.99; 1.46 | 0.0576 |  |
|  |  | 5 | 0.79 | 0.65; 0.97 | 0.023 |  |
|  |  | 6 | 0.86 | 0.73; 1.02 | 0.0823 |  |
|  |  | 7,1 | 0.96 | 0.81; 1.14 | 0.6657 |  |
|  |  | 9 | 1 | 0.78; 1.30 | 0.9788 |  |
|  |  | Others | 0.92 | 0.62; 1.36 | 0.6721 |  |
| IgG | VSA | 2 | 0.98 | 0.69; 1.40 | 0.9202 | 0.0044 |
|  |  | 3 | 0.87 | 0.60; 1.25 | 0.4369 |  |
|  |  | 4 | 1.02 | 0.74; 1.39 | 0.9146 |  |
|  |  | 5 | 0.76 | 0.56; 1.02 | 0.0694 |  |
|  |  | 6 | 0.67 | 0.51; 0.87 | 0.0032 |  |
|  |  | 7,1 | 0.76 | 0.57; 1.00 | 0.0478 |  |
|  |  | 9 | 0.76 | 0.52; 1.11 | 0.1501 |  |
|  |  | Others | 0.6 | 0.38; 0.95 | 0.0302 |  |

^1^ Proportional difference; ^2^ Confidence interval; ^3^P value using likelihood ratio test
