# Supplementary figures and images for: Impact of age of first exposure to Plasmodium falciparum on antibody responses to malaria in children: a randomized, controlled trial in Mozambique
Source: Malar J. 2014 Mar 27;13:121. doi: 10.1186/1475-2875-13-121 (PMC3986595; doi:10.1186/1475-2875-13-121)

Additional file 4


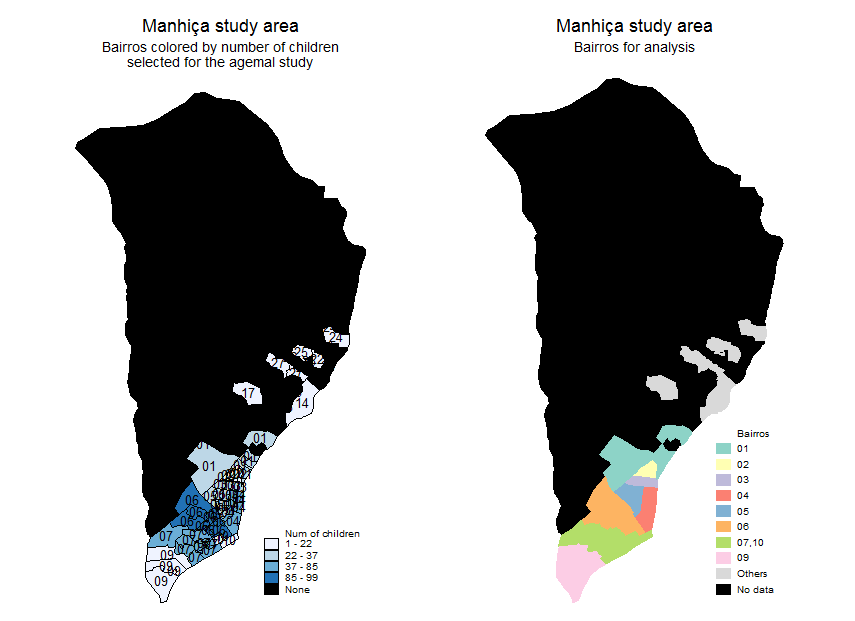


Bairros = Local terminology for Neighbourhoods

Num: Number

Supplement: Additional file 4 — District map of the Manhiça study area. Group of study regions and distribution of study participants per neighbourhood (bairros). [file 1475-2875-13-121-S4.doc]
