# Supplementary material for: Impact of age of first exposure to Plasmodium falciparum on antibody responses to malaria in children: a randomized, controlled trial in Mozambique
Source: Malar J. 2014 Mar 27;13:121. doi: 10.1186/1475-2875-13-121 (PMC3986595; doi:10.1186/1475-2875-13-121)
Supplement: Additional file 5 — Magnitude of antibody response related to incidence of clinical malaria only in control group. Association between levels of antibodies (2-fold increment) at 2.5 months of age and the incidence of malaria up to 12 and 24 months of age, only for the placebo control group. Analysis done by negative binomial regression models adjusted by season, neighborhood, current infection, previous infection, maternal infection, congenital infection, placental inflammation, insecticide treated bednet use and indoor residual spraying. [file 1475-2875-13-121-S5.doc]

Additional file 5

|  |  | 2.5-12 months | | | | | | 2.5-24 months | | | | | |
| --- | --- | --- | --- | --- | --- | --- | --- | --- | --- | --- | --- | --- | --- |
| Antigen | Antibody | Crude | | | Adjusted | | | Crude | | | Adjusted | | |
|  |  | IRR^1^ | 95% CI^2^ | P value^3^ | IRR | 95% CI | P value | IRR | 95% CI | P value | IRR | 95% CI | P value |
| MSP-1_19_ | IgG | 1.39 | 0.92;2.09 | 0.1147 | 1.38 | 0.93; 2.04 | 0.1200 | 1.21 | 0.86;1.71 | 0.2614 | 1.27 | 0.93;1.75 | 0.1350 |
|  | IgG1 | 1.40 | 0.97;2.01 | 0.0707 | 1.36 | 0.97;1.90 | 0.0901 | 1.25 | 0.91;1.75 | 0.1579 | 1.04 | 0.78;1.41 | 0.7755 |
|  | IgG2 | 2.67 | 0.85;8.42 | 0.0761 | 1.56 | 0.46;5.30 | 0.5111 | 1.69 | 0.68;4.17 | 0.2323 | 0.91 | 0.36;2.29 | 0.8391 |
|  | IgG3 | 1.57 | 0.63;3.92 | 0.3294 | 2.01 | 0.74;5.47 | 0.1740 | 1.44 | 0.76;2.75 | 0.2719 | 1.89 | 1.00;3.55 | 0.0475 |
|  | IgG4 | 3.03 | 0.42;21.90 | 0.2718 | 2.52 | 0.32;20.10 | 0.4039 | 2.12 | 0.52;8.60 | 0.3001 | 1.56 | 0.36;6.72 | 0.5497 |
|  | IgM | 3.67 | 0.87;15.55 | 0.0615 | 1.91 | 0.75;4.86 | 0.1699 | 1.84 | 0.82;4.12 | 0.1475 | 1.23 | 0.64;2.37 | 0.5264 |
| AMA-1 | IgG | 1.72 | 0.90;3.30 | 0.0848 | 1.43 | 0.77;2.66 | 0.2131 | 2.13 | 1.35;3.38 | 0.0016 | 1.37 | 0.91;2.05 | 0.1340 |
|  | IgG1 | 1.81 | 1.01;3.23 | 0.0356 | 1.69 | 0.92;3.09 | 0.0650 | 1.84 | 1.26;2.70 | 0.0023 | 1.22 | 0.86;1.75 | 0.2668 |
|  | IgG2 | 1.94 | 0.80;4.68 | 0.1347 | 1.31 | 0.65;2.61 | 0.4398 | 1.59 | 0.91;2.78 | 0.1130 | 1.14 | 0.68;1.92 | 0.6096 |
|  | IgG3 | 1.51 | 1.60;2.17 | 0.0210 | 1.05 | 0.70;1.57 | 0.8245 | 1.35 | 0.97;1.87 | 0.0522 | 1.06 | 0.79;1.42 | 0.7162 |
|  | IgG4 | 1.83 | 0.97;3.47 | 0.0586 | 0.85 | 0.45;1.61 | 0.6065 | 1.58 | 0.93;2.70 | 0.0803 | 0.71 | 0.38;1.34 | 0.2973 |
|  | IgM | 1.30 | 0.58;2.92 | 0.5288 | 0.57 | 0.29;1.14 | 0.1048 | 1.17 | 0.67;2.06 | 0.5783 | 0.73 | 0.41;1.30 | 0.2818 |
| EBA-175 | IgG | 1.41 | 0.88;2.24 | 0.1494 | 1.32 | 0.84;2.07 | 0.2130 | 1.11 | 0.81;151 | 0.5266 | 0.98 | 0.72;1.34 | 0.9152 |
|  | IgG1 | 1.40 | 0.90;2.16 | 0.1251 | 1.29 | 0.98;1.70 | 0.0766 | 1.26 | 0.93;1.72 | 0.1278 | 1.21 | 0.95;1.53 | 0.118 |
|  | IgG2 | 2.35 | 0.68;8.17 | 0.1681 | 1.32 | 0.29;5.98 | 0.7250 | 1.30 | 0.53;3.16 | 0.5574 | 0.71 | 0.28;1.80 | 0.4716 |
|  | IgG3 | 1.25 | 0.75;2.08 | 0.3909 | 1.70 | 1.03;2.79 | 0.0412 | 1.02 | 0.70;1.47 | 0.9211 | 1.14 | 0.80;1.62 | 0.4635 |
|  | IgG4 | 2.25 | 0.89;5.66 | 0.0796 | 1.48 | 0.56;3.87 | 0.4493 | 1.37 | 0.75;2.48 | 0.3120 | 0.85 | 0.43;1.68 | 0.6464 |
|  | IgM | 0.99 | 0.42;2.37 | 0.9894 | 1.15 | 0.48;2.75 | 0.7563 | 0.68 | 0.33;1.18 | 0.1527 | 0.63 | 0.33;1.19 | 0.1474 |
| VSA | IgG | 1.64 | 0.93;2.90 | 0.0855 | 1.52 | 0.97;2.38 | 0.0719 | 1.55 | 1.01;2.39 | 0.0488 | 1.28 | 0.90;1.81 | 0.1647 |

^1^Incidence rate ratio

^2^Confidence Interval

^3^Negative binomial regression model using likelihood ratio test
