# Supplementary material for: Impact of age of first exposure to Plasmodium falciparum on antibody responses to malaria in children: a randomized, controlled trial in Mozambique
Source: Malar J. 2014 Mar 27;13:121. doi: 10.1186/1475-2875-13-121 (PMC3986595; doi:10.1186/1475-2875-13-121)
Supplement: Additional file 6 — Breadth of antibody response related to incidence of clinical malaria only in control group. Analysis done by negative binomial regression. Participants of control group were assessed in five risk intervals: from 2.5 months to 12 months, from 2.5 months to 24 months, from 5.5 months to 24 months, from 10.5 to 24 months and from 15.5 to 24 months. The strength of the association between breadth of response and malaria risk was assessed first unadjusted and after adjusting by treatment, age, season, neighbourhood, current infection, previous infection, maternal infection, congenital infection, placental inflammation, insecticide treated bednet use and indoor residual spraying. [file 1475-2875-13-121-S6.doc]

| Control group | | | | | | | | | | | |
| --- | --- | --- | --- | --- | --- | --- | --- | --- | --- | --- | --- |
| Abs^1^ | Time | Crude | | | | | Adjusted | | | | |
|  |  | Bth^2^ | N^3^ | IRR^4^ | 95% CI^5^ | P value^6^ | Bth | N | IRR | 95% CI | P value |
| IgG | 2.5-12 months | 0-1 | 20 | 1 | _ | 0.1758 | 0-1 | 20 | 1 | _ | 0.2858 |
|  |  | 2 | 41 | 1.76 | 0.33;9.30 |  | 2 | 41 | 0.85 | 0.18;4.00 |  |
|  |  | 3 | 7 | 1.95 | 0.17;22.85 |  | 3 | 7 | 1.96 | 0.23;16.44 |  |
|  |  | 4 | 4 | 13.92 | 1.12;173.71 |  | 4 | 4 | 1.98 | 0.21;18.90 |  |
|  | 2.5-24 months | 0-1 | 20 | 1 | _ | 0.0749 | 0-1 | 20 | 1 | _ | 0.3076 |
|  |  | 2 | 41 | 3.48 | 1.06;11.41 |  | 2 | 41 | 1.07 | 0.38;2.99 |  |
|  |  | 3 | 7 | 2.46 | 0.40;15.27 |  | 3 | 7 | 2.37 | 0.49;11.52 |  |
|  |  | 4 | 4 | 12.09 | 1.47;99.61 |  | 4 | 4 | 2.60 | 0.47;14.32 |  |
|  | 5.5-24 months | 0-1 | 59 | 1 | _ | 0.0835 | 0-1 | 59 | 1 | _ | 0.5518 |
|  |  | 2 | 12 | 3.87 | 1.09;13.81 |  | 2 | 12 | 1.59 | 0.62;4.11 |  |
|  |  | 3 | 2 | 2.60 | 0.15; 45.97 |  | 3 | 2 | 1.80 | 0.29;11.14 |  |
|  | 10.5-24 months | 0-1 | 65 | 1 | _ | 0.8948 | 0-1 | 65 | 1 | _ | 0.2442 |
|  |  | 3 | 1 | 1.34 | 0.02;111.90 |  | 3 | 1 | 9.90E+08 | 0 |  |
|  | 15-24 months | 0-1 | 67 | 1 | _ | 0.0006 | 0-1 | 67 | 1 | _ | <0.0001 |
|  |  | 2 | 4 | 14.23 | 3.00;67.44 |  | 2 | 4 | 11.5 | 3.58;36.98 |  |
|  |  | 3 | 2 | 8.96 | 1.01;79.43 |  | 3 | 7 | 25.85 | 0.70;951.89 |  |
| IgM | 2.5-12 months | 0 | 93 | 1 | _ | 0.3049 | 0 | 93 | 1 | _ |  |
|  |  | 1 | 8 | 2.58 | 0.39;17.04 |  | 1 | 8 | 1.57 | 0.29;8.57 | 0.5946 |
|  | 2.5-24 months | 0 | 93 | 1 | _ | 0.1888 | 0 | 98 | 1 | _ | 0.3271 |
|  |  | 1 | 8 | 2.48 | 0.56;10.87 |  | 1 | 8 | 1.82 | 0.55;6.05 |  |
|  | 5.5-24 months | 0 | 86 | 1 | _ | 0.3812 | 0 | 86 | 1 | _ | 0.2671 |
|  |  | 1 | 11 | 1.81 | 0.45;7.31 |  | 1 | 11 | 2.16 | 0.56;8.35 |  |
|  | 10.5-24 months | 0 | 77 | 1 | _ | 0.8238 | 0 | 77 | 1 | _ | 0.3313 |
|  |  | 1 | 8 | 0.55 | 0.09;3.41 |  | 1 | 8 | 3.55 | 0.64;19.81 |  |
|  |  | 2 | 1 | 1.03 | 0.01;99.39 |  | 2 | 1 | 0.68 | 0.04;12.35 |  |
|  | 15-24 months | 0 | 84 | 1 | _ | 0.6790 | 0 | 84 | 1 | _ | 0.1149 |
|  |  | 1 | 4 | 0.26 | 0.01;5.06 |  | 1 | 4 | 0.15 | 0.01;1.89 |  |
|  |  | 2 | 3 | 1.37 | 0.08;22.49 |  | 2 | 3 | 2.89 | 0.50;16.68 |  |

Additional file 6

^1^ Antibody types

^2^ Breadth of antibodies

^3^ Number of children

^4^ Incidence rate

^5^ Confidence interval

^6^ Negative binomial regression model using likelihood ratio test
